# A Comparative Study of Multiscale Sample Entropy and Hierarchical Entropy and Its Application in Feature Extraction for Ship-Radiated Noise

**DOI:** 10.3390/e21080793

**Published:** 2019-08-14

**Authors:** Weijia Li, Xiaohong Shen, Yaan Li

**Affiliations:** 1 School of Marine Science and Technology, Northwestern Polytechnical University, Xi’an 710072, China; 2 Key Laboratory of Ocean Acoustics and Sensing (Northwestern Polytechnical University), Ministry of Industry of Information Technology, Xi’an 710072, China

**Keywords:** underwater signal processing, feature extraction, multiscale sample entropy (MSE), hierarchical entropy (HE), ship-radiated noise

## Abstract

The presence of marine ambient noise makes it difficult to extract effective features from ship-radiated noise. Traditional feature extraction methods based on the Fourier transform or wavelets are limited in such a complex ocean environment. Recently, entropy-based methods have been proven to have many advantages compared with traditional methods. In this paper, we propose a novel feature extraction method for ship-radiated noise based on hierarchical entropy (HE). Compared with the traditional entropy, namely multiscale sample entropy (MSE), which only considers information carried in the lower frequency components, HE takes into account both lower and higher frequency components of signals. We illustrate the different properties of HE and MSE by testing them on simulation signals. The results show that HE has better performance than MSE, especially when the difference in signals is mainly focused on higher frequency components. Furthermore, experiments on real-world data of five types of ship-radiated noise are conducted. A probabilistic neural network is employed to evaluate the performance of the obtained features. Results show that HE has a higher classification accuracy for the five types of ship-radiated noise compared with MSE. This indicates that the HE-based feature extraction method could be used to identify ships in the field of underwater acoustic signal processing.

## 1. Introduction

Identification and classification of marine vehicles are important in the field of underwater signal processing, as they are of great value in the military and marine economy [1,2,3,4]. An important aspect of the ship classification problem is to extract effective features from received signals. Features extracted from a signal are the representation of part of the signal’s characteristics. Insufficient characteristic reflection will lead to low accuracy of classification. Therefore, there is a great need for the development of feature extraction methods in the field of underwater signal processing.

The traditional feature extraction method is based on the frequency domain. There are many studies devoted to extracting the spectral characteristics of signals, such as the analysis of the power spectral density of signals [5]. However, studies show that traditional methods have shortcomings and limitations in practical applications. For example, the traditional spectrum-based method is based on the assumption of the linearity of the signals, which means the features extracted using this method will miss the signal’s nonlinear characteristics [6]. In this paper, we use entropy as a feature extraction method, which is based on the time domain and quantifies the complexity of the signal as a feature.

Entropy, as a feature extraction method measuring the system’s complexity in the time domain, has been maturely applied to fault diagnosis and pathological signal detection [7,8,9,10,11]. Pincus proposed the concept of approximate entropy (AE) based on the theory of Shannon entropy in 1991 [12]. However, the AE has self-matching terms in the calculation, which leads to bias of the result. This kind of bias results in two disadvantages in the calculation of AE. One is that the computation of AE is overly dependent on the length of data, and the other is the lack of correlation between the AE result and the signal complexity. Thus, in 2000, Richman and Moorman proposed an improvement of the AE, which is the sample entropy (SE) [13]. It solved the consistency problem in AE, and in the subsequent research, the fast sample entropy was proposed, which simplified the SE’s calculation [14]. However, in pathological research, a single scale cannot illustrate the whole information carried in signals. To distinguish different kinds of pathological signals and calculate the complexity of interested signals more accurately, multiscale sample entropy (MSE) based on the coarse-graining process [15,16,17] and hierarchical entropy (HE) based on hierarchical decomposition [18,19] have been proposed. HE, as a method improving MSE, is capable of roller bearing fault diagnosis [20]. Compared with the MSE, which only considers the lower frequency components of signals in the calculation, HE that retains both the lower frequency and higher frequency components of signals can better recognize different pathological signals in practical applications.

The calculation of entropy on a single scale only takes into account the temporal information in the signal. Although it has many advantages such as simple calculation, sometimes it cannot reflect the complexity differences between different signals accurately. Many previous research works applied the coarse-graining process to entropy. This improvement can describe the complexity of signals at different scales. For example, Li proposed a method of extracting the features of ship-radiated noise combined with variational mode decomposition (VMD) and multiscale permutation entropy (MPE) in 2017 [21]. Yang combined VMD with fluctuation-based dispersion entropy [22]. Chen proposed a new method based on permutation entropy and coarse-graining [23]. Shashidhar applied MSE to weak signal detection problems [24]. All of the above studies have proven that entropy based on multiple scales has certain applicability in feature extraction of underwater acoustic signals. However, they did not consider the high-frequency components in the signal. In other words, much useful information may be missed at a high frequency. Meanwhile, research showed that the lower frequency components of ambient noise have increased over the past few decades [25]. This makes it more difficult to deal with the detection and feature extraction of ship-radiated noise. If the lower and higher frequency components of the signal can be separately analyzed when extracting the features, we may get more complete information contained in the signal.

In this paper, HE is used as a novel feature extraction method for ship-radiated noise. It has great advantages compared with methods such as MSE, preserving both the low-frequency and high-frequency components of the signal while performing multi-scale decomposition and calculating the complexity of the signals of interest. Hence, HE describes the signal characteristics more accurately. Several sets of simulation signals were used to compare the difference between HE and MSE in identifying different types of signals, verifying that the HE has good recognition ability, especially for signals with similar low-frequency components and different high-frequency components. For those signals with different low frequency components and similar high frequency components, since HE also considers the low-frequency components of the signal, the actual results are comparable to those of MSE. At the end of this paper, five different types of ship-radiated noises are presented, using SE, MSE, and HE for feature extraction, respectively. In order to compare the performance of the features extracted by different methods more clearly, we will pass the different features through a probabilistic neural network and criticize the performance of different features through the accuracy of classification.

The rest of this paper organized as follows: Section 2 introduces the concept of SE, MSE, and HE. In Section 3, the proposed method is applied to the simulated signal to show the properties of HE and MSE. In Section 4, five types of ship-radiated noise are given to reflect the difference between the two feature extraction methods. Finally, Section 5 is the conclusion.

## 2. Basic Theory

### 2.1. Sample Entropy

Sample entropy quantifies a system’s degree of regularity by calculating the negative natural logarithm of conditional probability. It was developed by Richman and Moorman in 2000. Compared with approximate entropy, sample entropy eliminates the bias caused by self-matching. Meanwhile, it also reduces the computational time. Given a time series {x(i):1≤i≤N}, *N* is the length of the original time series. {x(i):1≤i≤N} can be reconstructed into a set of sequences as follows: X(i)=[x(i),x(i+1),⋯,x(i+m−1)]:1≤i≤N−m+1, where *m* is the embedding dimension. According to *m* and N+m−1 sequences, which were obtained above, the distance d[X(i),X(j)] between any two vectors can be defined, abbreviated as $D^{m}\left(i\right)$:

(1) $$D^{m}\left(i\right)=d\left[X\left(i\right),X\left(j\right)\right]=max[|x\left(i+k\right)-x\left(j+k\right)|]:0\leq k\leq m-1;1\leq i,j\leq N-m+1;i\neq j.$$

Since the time series {x(i):1≤i≤N} has already been given, the standard deviation (SD) of the time series can be readily obtained. Set r=0.1SD∼0.25SD to be the threshold, with the distance d[X(i),X(j)]. The formula of $B_{i}^{m}\left(r\right)$ is given by:

(2) $$B_{i}^{m}\left(r\right)=\frac{1}{N-m}\left\{\mathrm{the}\;\mathrm{number}\;\mathrm{of}\;d\left[X\left(i\right),X\left(j\right)\right]\leq r\right\}.$$

Equation (2) computes the probability of the distance between X(i) and the remaining sequences within the threshold *r*. Moreover, the average of $B_{i}^{m}\left(r\right)$ can obtained by Equation (3):

(3) $$B^{m}\left(r\right)=\frac{1}{N-m+1}\sum_{i=1}^{N-m+1}B_{i}^{m}\left(r\right).$$

Increasing the embedding dimension *m* to m+1, then $B_{i}^{m+1}\left(r\right)$ can be analogously obtained by repeating the previous steps. Finally, the sample entropy (SampEn(m,r,N)) is given by the following equation:

(4) $$SampEn\left(m,r,N\right)=-ln[B^{m+1}\left(r\right)/B^{m}\left(r\right)].$$

**Figure 1:**
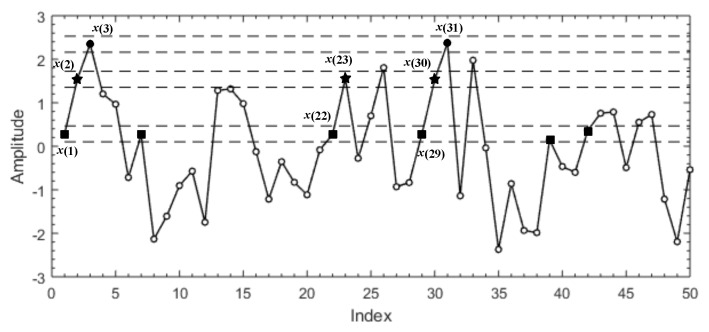
Stimulation signal.

In order to better understand the calculation process of sample entropy, we briefly describe it through Figure 1.

A time series {x(i):1≤i≤50} is given to illustrate the process for calculating SampEn(m,r,N). We specify m=2 and r=0.15SD. The horizontal dashed lines around x(1), x(2), and x(3) represent x(1)±r, x(2)±r, and x(3)±r, respectively. If the absolute difference between any two points is less than *r*, these two points match each other; also, it can be viewed as “indistinguishable”. In Figure 1, all of the points that match x(1), x(2), and x(3) are represented with same symbol, respectively. Let {x(1),x(2)} and {x(1),x(2),x(3)} be a template sequence with two points and three points, respectively. Throughout {x(i):1≤i≤50}, there are two sequences {x(22),x(23)} and {x(29),x(30)} that match the template sequence {x(1),x(2)}. As for template sequence {x(1),x(2),x(3)}, there is only one sequence {x(29),x(30),x(31) that matches it. Count the number of the sequences that match {x(1),x(2)} and {x(1),x(2),x(3)}. Repeat the previous steps for the next two-point sequence {x(2),x(3)} and three-point sequence {x(2),x(3),x(4)}. Sum the number of sequences that match two-point and three-point sequence {x(2),x(3)} and {x(2),x(3),x(4)}. Add them to the previous values that we already obtained. Repeat the same work mentioned above until all other possible template sequences ({x(1),x(2),x(3)},⋯,{x(48),x(49),x(50)}) are considered. The ratio between the sum of two-point template matches and the sum of three-point template matches can be obtained. Therefore, SampEn(m,r,N) is the natural logarithm of this ratio.

The value of SampEn(m,r,N) is related to the parameters *m* and *r*. Therefore, the choices of these two parameters are also very important. According to Chen’s research [26], *m* is set to be one or two, and r=0.1SD∼0.25SD under most circumstances.

### 2.2. Multiscale Sample Entropy

Although SE has many advantages, in some circumstances, it cannot reflect the complexity differences between different signals accurately. The structure of signals generated from complex systems exhibits multiple temporal scale characteristics in the actual ocean environment. SE, as a single-scale-based method, does not account for the interrelationship between entropy and multiple scales. In order to overcome this shortage, Costa et al. developed the concept of multiscale sample entropy [15]. MSE can be viewed as SE with a coarse-graining process for the time series [27]. The coarse-graining process is based on averaging the samples inside moving, but non-overlapping windows. For a given time series {x(i):1≤i≤N}, the coarse-graining process is denoted as:

(5) $$y^{\left(n\right)}=\frac{1}{n}\sum_{j=1}^{n}x\left(ni-n+j\right):1\leq i\leq N_{n},$$

where *N* is the length of the time series and $N_{n}=\left\lfloor \frac{N}{n}\right\rfloor$ stands for the largest integer no greater than $\frac{N}{n}$. Hence, MSE at scale *n* is obtained by calculating the sample entropy of $y^{\left(n\right)}$. The MSE focuses on lower frequency components of a time series. However, it ignores the information contained in the higher frequency components of the signal. This problem leads to the development of hierarchical entropy.

### 2.3. Hierarchical Entropy

Hierarchical entropy (HE) is an algorithm quantifies the “complexity” of a time series based on SE and hierarchical decomposition. Unlike MSE, hierarchical decomposition takes both higher and lower frequency components of a time series into consideration [18]. Specifically, for a given time series, $x=\{x\left(i\right):1\leq i\leq 2^{n}\}$. The definition of two operators $Q_{0}$ and $Q_{1}$ is as follows:

(6) $$Q_{0}\left(x\right)=\left(\frac{x(2i-1)+x(2i)}{2}:1\leq i\leq 2^{n-1}\right),$$

(7) $$Q_{1}\left(x\right)=\left(\frac{x(2i-1)-x(2i)}{2}:1\leq i\leq 2^{n-1}\right),$$

$Q_{0}\left(x\right)$ and $Q_{1}\left(x\right)$ are respectively the lower and higher frequency component of time series x, and their scale is two and their length $2^{n-1}$. As a matter of fact, x can be reconstructed from $Q_{0}\left(x\right)$ and $Q_{1}\left(x\right)$.

(8) $$x=Q_{0}{\left(x\right)}_{j}+Q_{1}{\left(x\right)}_{j},Q_{0}{\left(x\right)}_{j}-Q_{1}{\left(x\right)}_{j}:1\leq j\leq 2^{n-1}.$$

$Q_{0}{\left(x\right)}_{j}$ and $Q_{1}{\left(x\right)}_{j}$ stand for the $j^{\mathrm{th}}$ value in $Q_{0}\left(x\right)$ and $Q_{1}\left(x\right)$, respectively. Thus, $Q_{0}\left(x\right)$ and $Q_{1}\left(x\right)$ constitute the two-scale hierarchical decomposition of the time series x.

**Figure 2:**
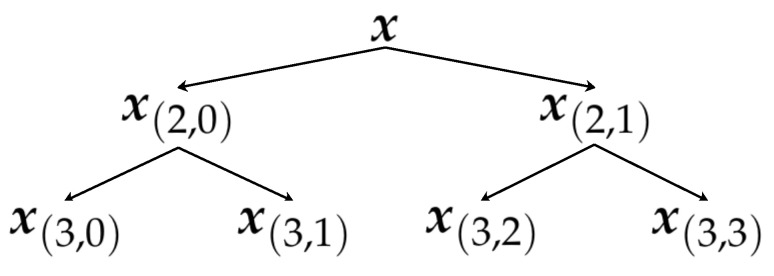
Hierarchical decomposition of the signal with three scales.

After we obtain $Q_{0}\left(x\right)$ and $Q_{1}\left(x\right)$, each of them can also be decomposed by $Q_{0}$ and $Q_{1}$. Consequently, we can get the hierarchical decomposition of the time series *X* at a scale of three. A tree graph can clearly show the relationship between each hierarchical component of the time series *X* in Figure 2.

After the hierarchical decomposition, several sub-signals $x_{\left(n,e\right)}$ can be obtained, where *n* represents the scale and *e* stands for the $e^{\mathrm{th}}$ sub-signal at scale *n*. Calculate the SE for each sub-signal, and the HE result of *X* is obtained. It is important to choose the appropriate scales in different circumstances. On the one hand, high scales usually lead to computational redundancy. On the other hand, low scales may have insufficient accuracy in SampEn(m,r,N)’s computation.

## 3. Simulation Analysis of Different Signals Based on Hierarchical Entropy and Multiscale Sample Entropy

In this section, MSE and HE are compared using different simulation signals in order to illustrate their different characteristics. Before the simulation analysis, there are some previous steps that need to be done. In this paper, all the SE calculation’s parameters are the same, which is m=2, r=0.15SD, and the length of the data is at least 512 points for every SE calculation. In this part, the content is divided into the following subsections. First, we prove that the parameters chosen when calculating SE are appropriate. Second, three different orders of AR signals with different complexity are used to prove that HE is an effective measure of complexity. Third, different simulation signals are constructed, and their results of HE and MSE are compared. The results show that MSE pays more attention to the low-frequency components of the signal, and HE not only retains the information of the low-frequency components of the signal, but also retains the information of the high-frequency components of the signal. Finally, considering the noise interference in practical applications, this paper compares the robustness of the two methods to noise.

### 3.1. Parameter Selection for Sample Entropy

Both HE and MSE are based on SE. When we calculate the SE for a signal, it is important to choose the appropriate *m* and *r*. Since our main purpose is using entropy as a feature extraction method for ship-radiated noise, the simulation signals in this subsection are set as follows:

(9) $$\left\{\begin{array}{c} S_{1}\left(n\right)=sin\left(2\pi *50n\right)+N\left(n\right),\\ S_{2}\left(n\right)=sin\left(2\pi *13n\right)+N\left(n\right), \end{array}\right.$$

**Figure 3:**
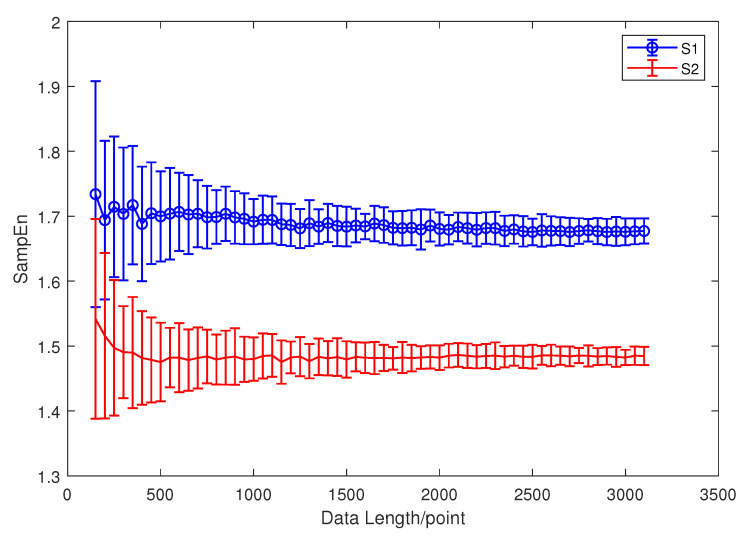
$S_{1}\left(n\right)$ and $S_{2}\left(n\right)$’s SE results with different lengths of data.

In Equation (9), $S_{1}\left(n\right)$ and $S_{2}\left(n\right)$ are two sinusoidal signals mixed with Gaussian white noise. We use the sinusoidal signal in order to simulate the periodic signal produced by the ship engine or propeller. Meanwhile, Gaussian white noise is used to simulate the ambient noise. Since the composition of the ship-radiated noise is very complex, including ambient noise, cavitation noise, and signals produced by propellers and the engine, we simplify the model of ship-radiated noise as Equation (9). The signal-to-noise ratio (SNR) is set to be 5 dB, m=2, and r=0.15SD. To demonstrate the impact of different data lengths on the calculation results, we calculated 60 sets of SE results with different lengths of the two signals, each with 30 results. The data length increased from 150 equal intervals to 3150. The result is shown in Figure 3.

In Figure 3, as the length of the calculated sample entropy data increases, the results of the calculation become gradually stable. When the data length is too short, the SE results are too unstable to distinguish the sinusoidal signals of two different frequencies very well. Although the result becomes more stable as the data length increases, due to the consideration of the amount of calculation, when calculating the sample entropy in the paper, the data length is unified to 512. When we calculate HE in this paper, since the data length is 8192 points, we decompose the signal into a scale of five and guarantee that the SE’s calculation that is contained in HE is at least 512 points.

**Figure 4:**
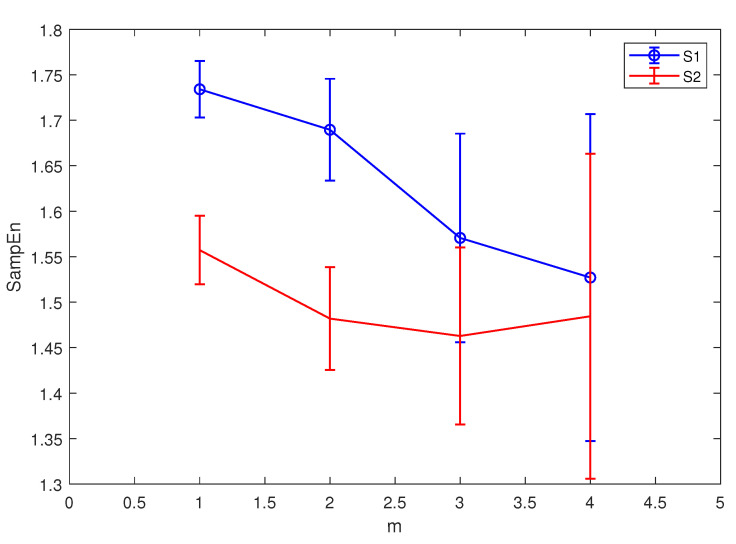
$S_{1}\left(n\right)$’s and $S_{2}\left(n\right)$’s SE results with different *m*.

**Figure 5:**
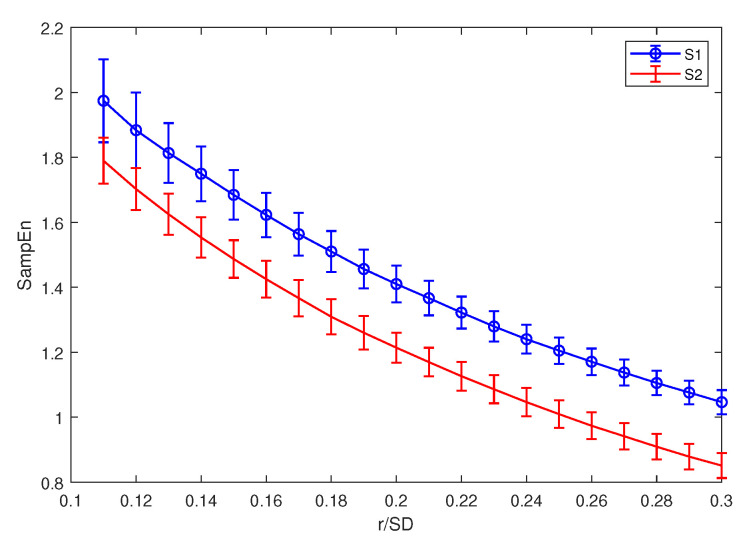
$S_{1}\left(n\right)$’s and $S_{2}\left(n\right)$’s SE results with different *r*.

After selecting the appropriate data length, the same simulated signals in Equation (9) are used to choose the value of *m* and *r*. The length of signal is set to be 512 points when calculating SE. The result is displayed in Figure 4 and Figure 5.

**Figure 6:**
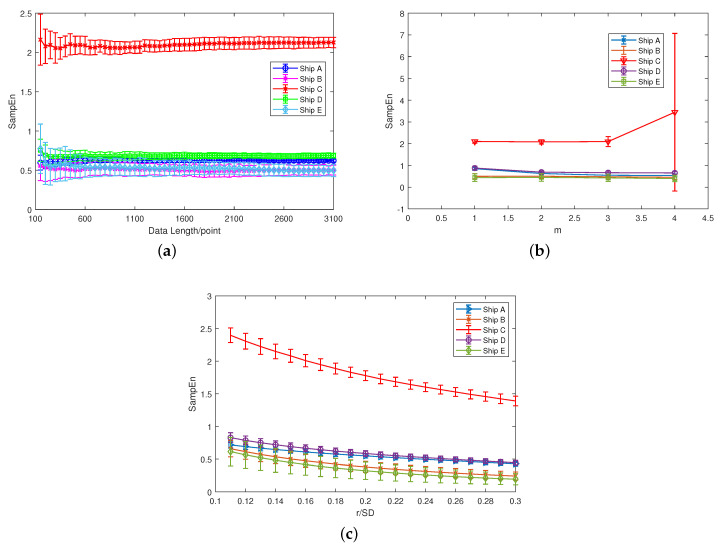
SE results for five types of ship-radiated noise with different parameters. (**a**) SE results with different data length. (**b**) SE results with different *m*. (**c**) SE results with different *r*.

From Figure 4 and Figure 5, the result of SE is too close to distinguish two signals when m=3, and it becomes unstable when *m* is larger than three, so we set m=2 in this paper. As for *r*, the value of *r* has little effect on the stability of the results, so we set r=0.15∗SD. The same parameters are discussed using the real ship-radiated noise employed in this paper [28], further verifying the conclusion in this section. The results are demonstrated in Figure 6. For some certain types of ship-radiated noise, SE cannot distinguish them very well according to Figure 6. This is why we need to introduce HE as a new feature extraction method to help us distinguish different signals.

### 3.2. Hierarchical Entropy Analysis for the AR Process

Three autoregressive processes (AR) with different orders will be given to demonstrate that HE is an effective method for measuring the complexity of different signals. The AR time series are given by:

(10) $$AR_{p}\left(t\right)=\sum_{i=1}^{p}\alpha_{i}AR\left(t-i\right)+n\left(t\right),$$

where n(t) is the Gaussian white noise with a standard normal distribution. The length of each AR process is $2^{13}$. *p* indicates the order of the AR process, and $\alpha_{i}$ is the correlation coefficients. The value of $\alpha_{i}$ in each AR process is given in Table 1 according to [29].

**Figure 7:**
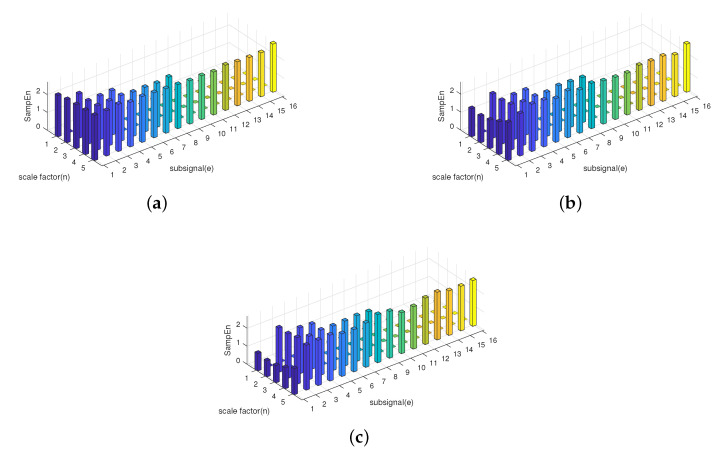
Hierarchical entropy results of AR(1), AR(4), and AR(7). (**a**) HE results for AR(1). (**b**) HE results for AR(4). (**c**) HE results for AR(7).

The HE results of three AR time series are illustrated in Figure 7; HE(n,e) stands for the $e^{\mathrm{th}}$ component of hierarchical entropy at scale *n*, and this abbreviation is used throughout this paper.

The AR process specifies that the output value is linearly dependent on its own previous and random terms. The dependence of the output value on the previous terms increases as the order *p* increases. Furthermore, as the order *p* increases, the correlation of the signal increases accordingly, making the model more predictable [23,29]. That is, the complexity of AR(p+1) is lower than that of AR(p). Based on this idea, the value of HE should be negatively correlated to order *p*. Figure 7 depicts that the sample entropy of lower frequency components decreases while the order *p* of the AR time series increases. Hence, HE can be confirmed as an effective method for measuring the complexity of different time series.

### 3.3. Properties for Multiscale Sample Entropy

In this section, a set of simulation signals is employed to demonstrate the properties for MSE, which is focused on the lower frequency components of the signal. This property leads to the result that MSE performs well in distinguishing the signals with different low-frequency components. In order to highlight these properties of MSE, a set of signals is given as follows:

(11) $$f_{1}\left(n\right)=\left\{\begin{array}{c} sin\left(2\pi *5n\right):1\leq n\leq \left(2^{13}-2^{10}\right),\\ sin\left(2\pi *60n\right):\left(2^{13}-2^{10}\right)+1\leq n\leq 2^{13}. \end{array}\right.$$

(12) $$f_{2}\left(n\right)=\left\{\begin{array}{c} sin\left(2\pi *15n\right):1\leq n\leq \left(2^{13}-2^{10}\right),\\ sin\left(2\pi *60n\right):\left(2^{13}-2^{10}\right)+1\leq n\leq 2^{13}. \end{array}\right.$$

**Figure 8:**
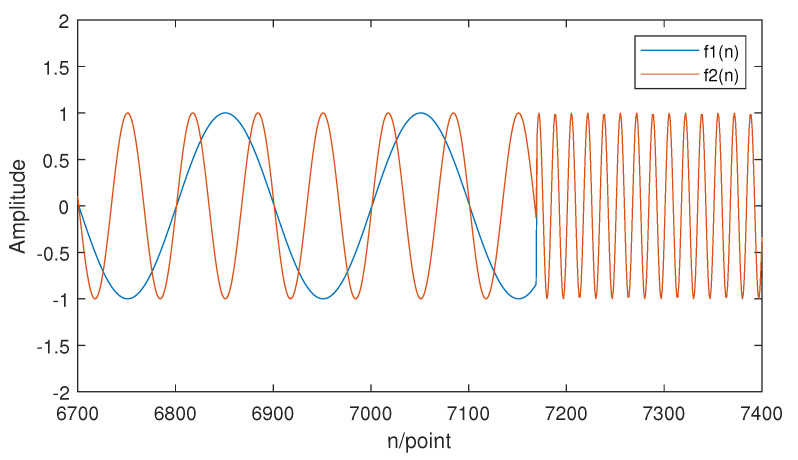
The waveform of $f_{1}\left(n\right)$ and $f_{2}\left(n\right)$.

**Figure 9:**
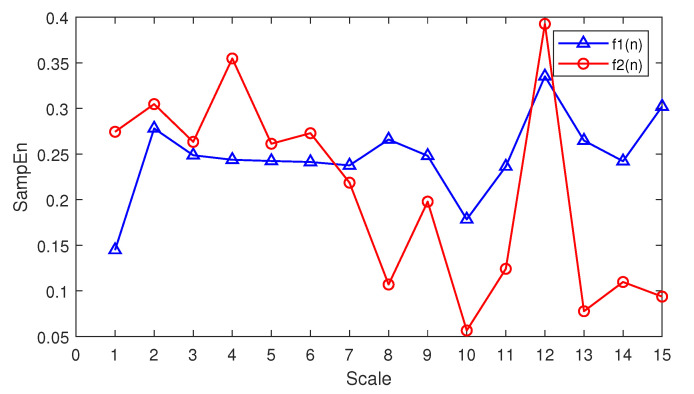
The MSE result for $f_{1}\left(n\right)$ and $f_{2}\left(n\right)$ at a scale of 1∼15.

The lower frequency components of $f_{1}\left(n\right)$ and $f_{2}\left(n\right)$ are different, while the high-frequency components are the same. The waveform of $f_{1}\left(n\right)$ and $f_{2}\left(n\right)$ is shown in Figure 8. According to the theory of MSE, MSE should be able to distinguish between the two signals very well since the difference between the two signals is mainly in the lower frequency components. Figure 9 is the MSE result for $f_{1}\left(n\right)$ and $f_{2}\left(n\right)$ from a scale of 1–15.

$f_{1}\left(n\right)$ and $f_{2}\left(n\right)$ can be distinguished by MSE easily since the two signals’ MSE have a great difference when the scale is greater than eight. Therefore, MSE performs well when distinguishing signals with different low-frequency components.

### 3.4. Properties for Hierarchical Entropy

According to the basic theory of hierarchical entropy, it takes into account higher frequency components of the signal when calculating, while sample entropy and multiscale sample entropy do not. Consequently, hierarchical entropy performs better when measuring the complexity of those signals whose information is stored in both lower and higher frequency components. In order to illustrate this characteristic, a set of synthetic signals are given as follows:

(13) $$f_{3}\left(n\right)=\left\{\begin{array}{c} sin\left(2\pi *5n\right):1\leq n\leq \left(2^{13}-2^{10}\right),\\ sin\left(2\pi *60n\right):\left(2^{13}-2^{10}\right)+1\leq n\leq 2^{13}. \end{array}\right.$$

(14) $$f_{4}\left(n\right)=\left\{\begin{array}{c} sin\left(2\pi *5n\right):1\leq n\leq \left(2^{13}-2^{10}\right),\\ sin\left(2\pi *50n\right):\left(2^{13}-2^{10}\right)+1\leq n\leq 2^{13}. \end{array}\right.$$

**Figure 10:**
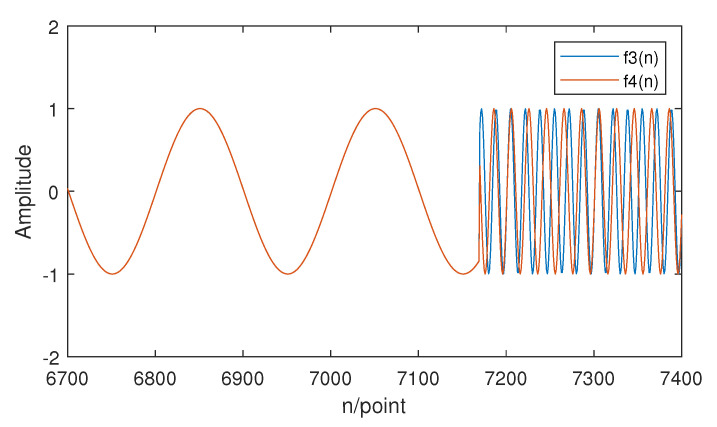
The waveform of $f_{3}\left(n\right)$ and $f_{4}\left(n\right)$.

$f_{3}\left(n\right)$ and $f_{4}\left(n\right)$ are signals that contain both higher and lower frequency components. Part of the waveform of $f_{3}\left(n\right)$ and $f_{4}\left(n\right)$ is shown in Figure 10.

**Figure 11:**
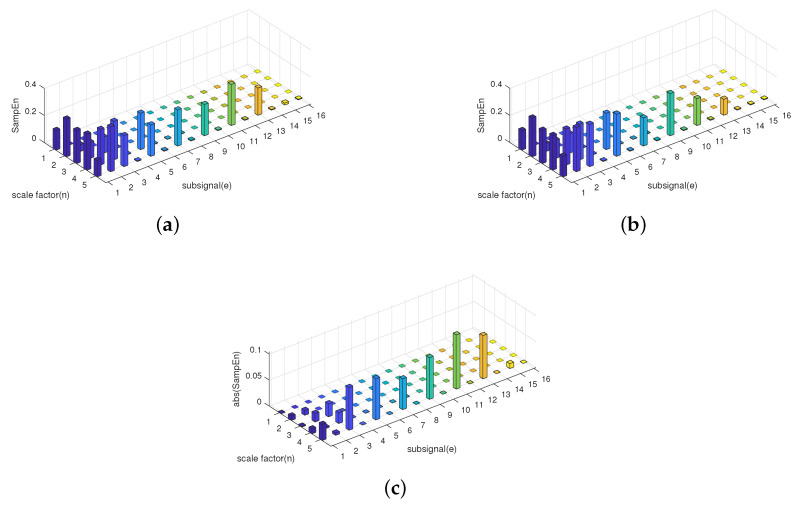
Hierarchical entropy results of $f_{3}\left(n\right)$ and $f_{4}\left(n\right)$. (**a**) HE results for $f_{3}\left(n\right)$. (**b**) HE results for $f_{4}\left(n\right)$. (**c**) HE’s absolute difference.

It is obvious that the information stored in the lower frequency components is the same, while the information stored in the higher frequency components is different. Based on the theory of sample entropy and multiscale sample entropy, only the lower frequency part is considered, which will lead to lower accuracy in distinguishing different signals while using SE or MSE. However, HE still measures the complexity of $f_{3}\left(n\right)$ and $f_{4}\left(n\right)$ very well since it considers the information stored in the higher frequency component. The HE results of two signals is displayed in Figure 11. The numerical result of SE, MSE, and HE is also shown in Table 2.

Before the interpretation of the results, first, some abbreviations are explained. MSE(*i*) stands for the multiscale entropy of signals at scale *i*, and HE(n,e) stands for the $e^{\mathrm{th}}$ component of the hierarchical entropy at scale *n*. These abbreviation are also used in the rest of this paper. According to the results displayed in Figure 11 and Table 2. The histogram at a scale of one is the sample entropy of the signal, HE(i,0) is equivalent to MSE($2^{i-1}$). Based on this equivalence relationship between MSE and HE, the HE results of $f_{3}\left(n\right)$ and $f_{4}\left(n\right)$ illustrated in Figure 11 also include part of the results of MSE. From Figure 11c, the HE results of the low frequency components of the two signals are not much different, but in some of the high-frequency components, the two signals can be successfully distinguished. That is to say, MSE cannot distinguish between signals that differ only in high-frequency components. Hence, HE has a better performance than SE or MSE in distinguish different frequency signals, especially when the information of the signal is mainly stored in higher frequency components.

## 4. Feature Extraction of Ship-Radiated Noise Based on Hierarchical Entropy

### 4.1. Feature Extraction Method Based on HE

**Figure 12:**
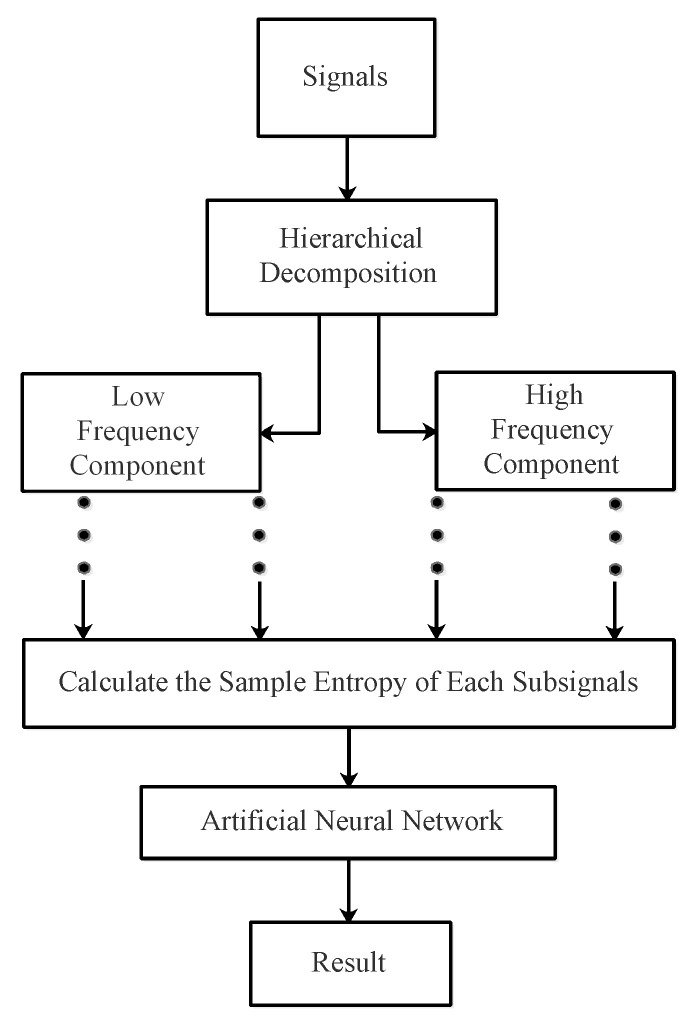
The flowchart of HE the feature extraction method.

The main steps of the feature extraction method based on HE are shown in Figure 12.

- Step 1: Five types of ship-radiated noise are given in this paper; choose the appropriate hierarchical decomposition order to guarantee that the length of sub-signal is longer than 512.
- Step 2: By doing the hierarchical decomposition *n* times, $2^{n}$ sub-signals can be obtained, representing the lower and higher frequency components of the original signal, respectively.
- Step 3: Calculate the sample entropy for each sub-signal. Get the HE result.
- Step 4: Flatten the HE matrix into a vector. Pass the vector through an artificial neural network.
- Step 5: Get the classification results.

### 4.2. Feature Extraction of Ship-Radiated Noise Based on HE

In this section, five types of ship-radiated noise were employed for the feature extraction (the ship-radiated noise of Ships D and E can be obtained from https://www.nps.gov/glba/learn/nature/soundclips.htm). The sampling frequency of Ships A, B, and C was 52.7 kHz. As for Ships D and E, the sampling frequency was 44.1 kHz. Ship A was a cruise ship. The vessel was less than 50 m away from the hydrophone. The hydrophone depth was 4.8 m. Ship B was an ocean liner. The vessel was less than 50 m away from the hydrophone. The hydrophone depth was 5.8 m. Ship C was a motorboat. The distance between the vessel and the hydrophone changed from 50 m–100 m during the recording of the data approximately.

**Figure 13:**
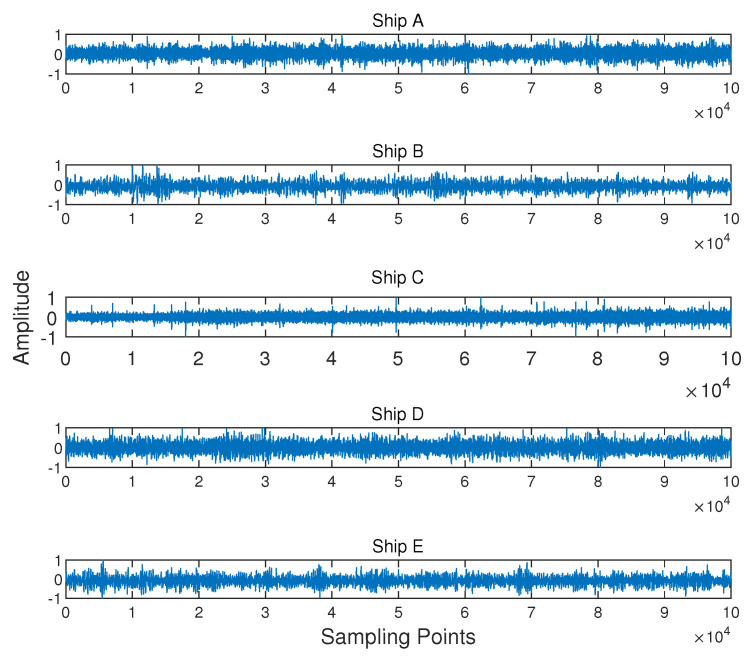
The waveform of the five types of ship-radiated noise.

**Figure 14:**
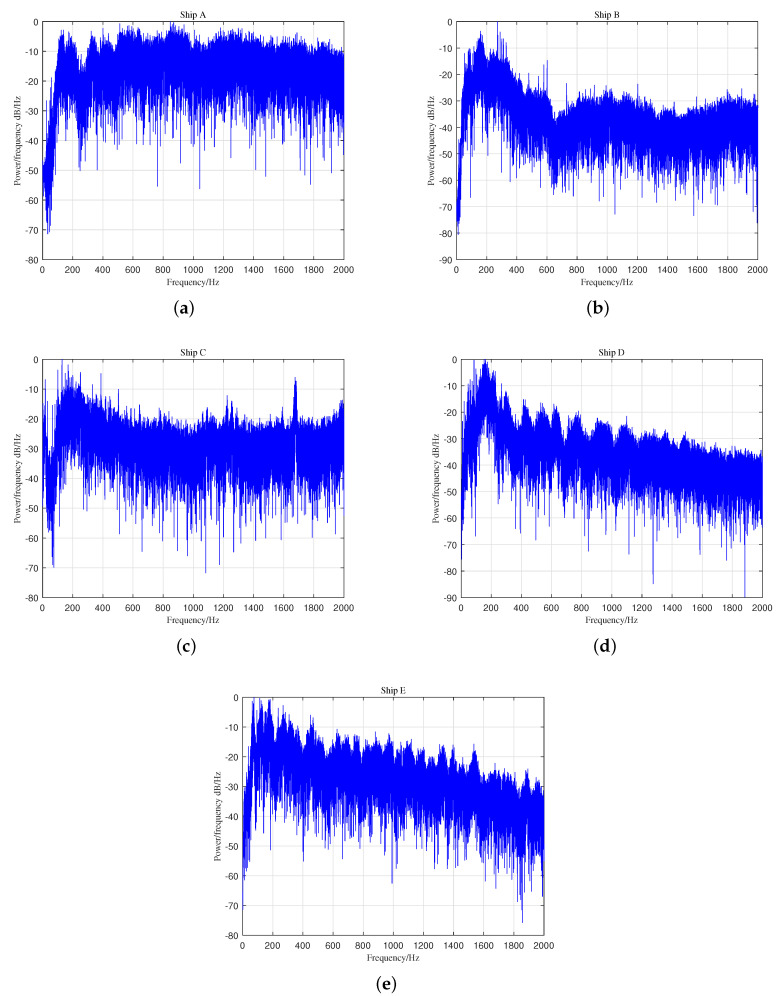
The power spectrum density analysis results of the five types of ship-radiated noise. (**a**) Ship A. (**b**) Ship B. (**c**) Ship C. (**d**) Ship D. (**e**) Ship E.

The hydrophone depth was 5.8 m. Further information for Ships A, B, and C can be found in [30]. Ships D and E were downloaded from a public website [31]. We chose a part of each signal and divided them into 100 segments separately. The length of each segment was 8192 sample points, namely 0.18 s of real-world data for Ships D and E and 0.15 s of real-world data for Ships A, B, and C. We can obtain 100 results for each type of ship-radiated noise by calculating the HE and MSE for every segment. The number of hierarchical decompositions was set as five. The waveform of five types of ship-radiated noise is demonstrated in Figure 13. Figure 14 gives the power spectrum density analysis results of the five types of signals.

Much useful information can be obtained from the power spectrum density analysis results of the five types of ship-radiated noise in Figure 14. The narrow-band spectral lines existing in Figure 14b,c make it easy to distinguish Ship B and Ship C. As for the rest of the types of ship, which are Ships A, D, and E in Figure 14a,d,e, few spectral lines can be found for us to distinguish different types of ship. Especially for Ships D and E, the fact that there was no evident distinction existing in their broadband spectral envelops made it difficult for us to distinguish these two types of ships accurately. Therefore, classifying these five different ships using the spectrum as a feature is difficult.

**Figure 15:**
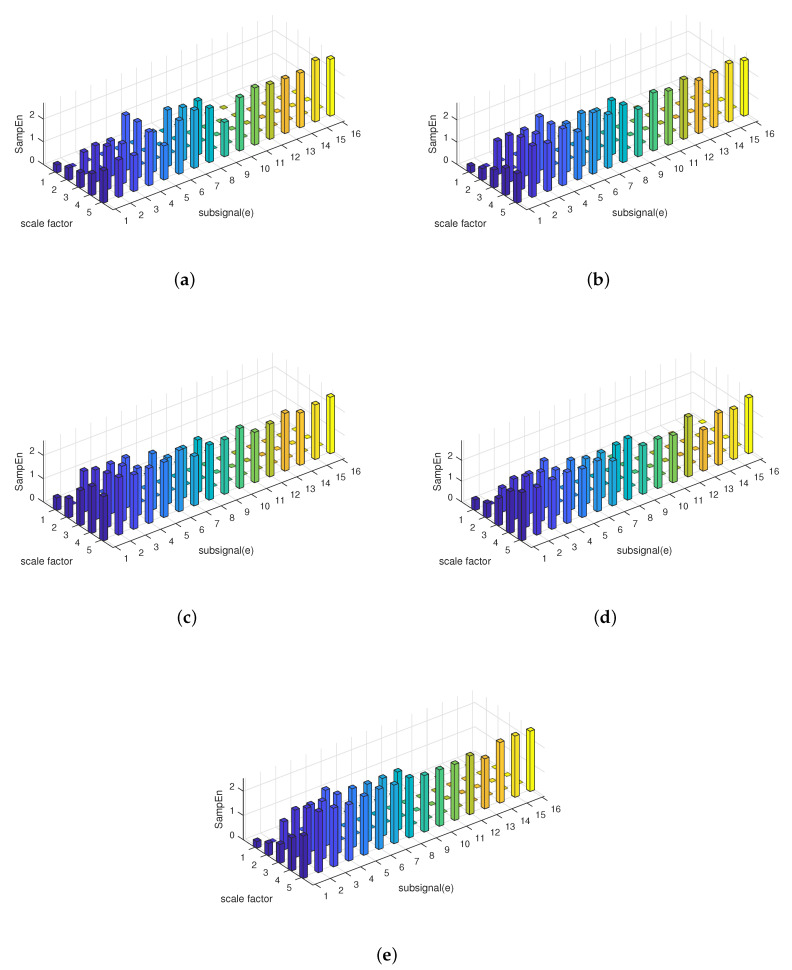
The HE results for the five types of ship-radiated noise. (**a**) Ship A. (**b**) Ship B. (**c**) Ship C. (**d**) Ship D. (**e**) Ship E.

**Figure 16:**
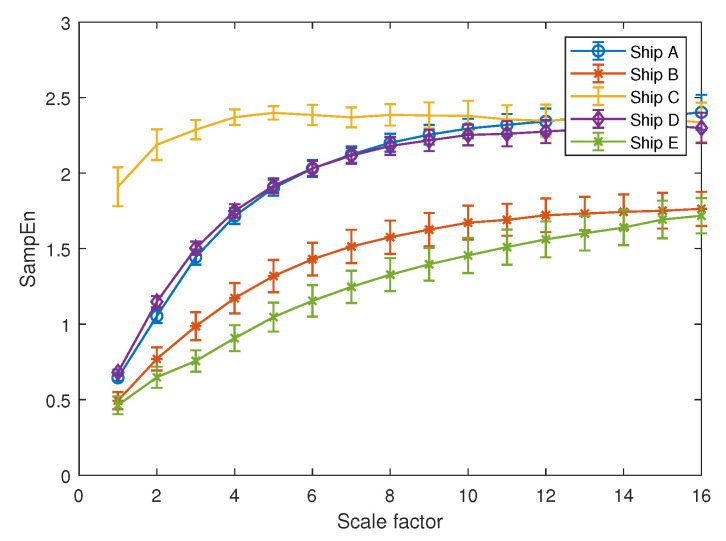
MSE results of the five types of ship-radiated noise.

The HE results of the five types of ship-radiated noise are illustrated in Figure 15. In order to compare the performance when HE and MSE both calculate the same data length for their sub-signals, Figure 16 shows the MSE result of the five types of ships from a scale of 1–16. Guarantee that when calculating the HE at a scale five, the length of the sub-signal was 512 points, the same as MSE at a scale of 16. Since it is difficult to see the differences between the five types of ship-radiated noise through Figure 15, part of the HE results are also shown numerically through Table 3. HE(n)represents the HE result at scale *n*.

According to the MSE result demonstrated in Figure 16, we can see that SE can only distinguish Ship C from other types of ship. Throughout the MSE result from a scale of 1–16, the entropy differences between Ships A and D and Ships B and E remained small.

To evaluate the performance of the above-mentioned feature extraction methods quantitatively, the results of two methods were separately classified and identified by a probabilistic neural network. Since the MSE’s results for the five types of ships were vectors of length 16, we fed the probabilistic neural network with these vectors to get the classification results. As for HE, we flattened the HE’s results from matrices into vectors of a length of 31, then fed the PNN with these vectors to get the classification results. The classification results are demonstrated in Table 4, Table 5 and Table 6. The training set for each type of ship was 70, and the test set was 30.

Before assessing the performance of the PNN, the definitions of “sensitivity” and “specificity” are given as follows:

(15) $$\left\{\begin{array}{c} Sensitivity=\frac{TP}{TP+FN},\\ Specificity=\frac{TN}{TN+FP}, \end{array}\right.$$

where TP, TN, FP, and FN are the abbreviations for “true positive”, “true negative ”, “false positive”, and “false negative”, respectively. It is important to note that “accuracy” calculates the overall classification accuracy of neural networks, which is also the average of “sensitivity”.

From Table 4, Table 5 and Table 6, it is obvious that HE was able to classify five types of ships very well. Even for those types of ships that SE and MSE could not classify, their sensitivities in HE’s result were very high. The accuracy of HE increased 9.3% compared with MSE and 23.3% compared with SE. In order to eliminate the impact of sampling frequency, we reduced the sampling frequency of Ships A, B, and C from 52.7 kHz to 44.1 kHz, calculated the HE results for five types of ships, and passed the results through PNN. The classification result is demonstrated in Table 7. Through the table, we can see that the classification accuracy was 96%, very close to the accuracy of not reducing the sampling frequency.

Moreover, we mixed five types of ship-radiated noise with Gaussian white noise. The SNR was set to be 5 dB, and the classification results are illustrated in Table 8 and Table 9.

According to the results shown in Table 8 and Table 9, as the noise mixed into the ship-radiated noise, both HE and MSE were affected. However, even though the accuracy of both methods decreased, HE’s accuracy remained higher compared with MSE. The accuracy of HE decreased by 5.3% with added noise, while the accuracy of MSE decreased by 14.7% under the same conditions. Furthermore, even when the ship-radiated noise was mixed with noise, HE could still distinguish Ship C very well.

## 5. Conclusions

A new method was proposed for feature extraction of ship-radiated noise based on hierarchical entropy in this paper. The simulation analysis indicated that HE had better performance compared with MSE when the differences between signals were mainly focused on their high-frequency components. Applying two feature extraction methods to ship-radiated noise could help distinguish some signals that were not very different in the frequency domain. Moreover, in order to compare the performance of HE and MSE, we passed the extracted features through a neural network, and the classification results showed that the classification accuracy of HE was higher than MSE. In summary, since HE considered more information, as a new feature extraction method in the field of underwater acoustic signal processing, HE can better distinguish different signals in most circumstances than traditional entropy-based methods such as MSE.

## Figures and Tables

**Table 1:** The correlation coefficients for generating AR processes.

	$\alpha_{1}$	$\alpha_{2}$	$\alpha_{3}$	$\alpha_{4}$	$\alpha_{5}$	$\alpha_{6}$	$\alpha_{7}$
AR(1)	0.5	-	-	-	-	-	-
AR(4)	0.5	0.25	0.125	0.0625	-	-	-
AR(7)	0.5	0.25	0.125	0.0625	0.0313	0.0156	0.0078

**Table 2:** Different entropy’s results of $f_{3}\left(n\right)$ and $f_{4}\left(n\right)$.

	SE	MSE(2)	MSE(4)	HE(5,9)	HE(5,13)
$f_{3}\left(n\right)$	1.1447	0.2769	0.2419	0.2320	0.1533
$f_{4}\left(n\right)$	1.1442	0.2862	0.2460	0.3102	0.2645
Absolute Difference	0.0005	0.0093	0.0041	0.0782	0.1112

**Table 3:** Part of HE results for five types of ship-radiated noise.

Ship Type	SE	MSE(2)	MSE(4)	MSE(8)	HE(3,3)	HE(4,7)	HE(5,3)	HE(5,13)
**Ship A**	0.64	1.04	1.72	2.21	2.25	2.19	2.08	2.17
**Ship B**	0.41	0.83	1.21	1.55	2.41	2.49	2.35	2.45
**Ship C**	1.92	2.13	2.23	2.37	2.36	2.45	2.41	2.51
**Ship D**	0.66	1.07	1.65	2.10	2.39	2.36	2.29	2.38
**Ship E**	0.42	0.75	1.06	1.53	2.37	2.47	2.72	2.61

**Table 4:** Probabilistic neural network classification results of SE.

Type	Recognized as					Sensitivity	Specificity
	A	B	C	D	E		
**A**	28	0	0	2	0	93.3%	90%
**B**	3	27	0	0	0	90%	75%
**C**	0	0	30	0	0	100%	100%
**D**	9	0	0	21	0	70%	96.7%
**E**	0	30	0	0	0	0%	100%
**Accuracy**	70.7%						

**Table 5:** Probabilistic neural network classification results of MSE(1)∼(16).

Type	Recognized as					Sensitivity	Specificity
	A	B	C	D	E		
**A**	21	0	0	9	0	70%	96.7%
**B**	0	25	0	0	5	83.3%	95.8%
**C**	0	0	30	0	0	100%	100%
**D**	4	0	0	26	0	86.7%	92.5%
**E**	0	5	0	0	25	83.3%	95.8%
**Accuracy**	84.7%						

**Table 6:** Probabilistic neural network classification results of HE(1)∼(5).

Type	Recognized as					Sensitivity	Specificity
	A	B	C	D	E		
**A**	25	0	0	5	0	83.3%	99.2%
**B**	0	27	0	0	3	90%	100%
**C**	0	0	30	0	0	100%	100%
**D**	1	0	0	29	0	96.7%	95.8%
**E**	0	0	0	0	30	100%	97.5%
**Accuracy**	94%						

**Table 7:** Probabilistic neural network classification results of HE(1)∼(5) after reducing the sampling frequency.

Type	Recognized as					Sensitivity	Specificity
	A	B	C	D	E		
**A**	27	0	0	3	0	90%	98.3%
**B**	0	29	0	0	1	96.7%	100%
**C**	0	0	30	0	0	100%	100%
**D**	2	0	0	28	0	93.3%	97.5%
**E**	0	0	0	0	30	100%	99.1%
**Accuracy**	96%						

**Table 8:** (Noise) Probabilistic neural network classification results of HE(1)∼(5).

Type	Recognized as					Sensitivity	Specificity
	A	B	C	D	E		
**A**	26	0	1	3	0	86.7%	97.5%
**B**	0	24	0	5	1	80%	97.5%
**C**	0	0	30	0	0	100%	99.2%
**D**	3	2	0	25	0	83.3%	92.5%
**E**	0	30	0	0	0	93.3%	99.2%
**Accuracy**	88.7%						

**Table 9:** (Noise) Probabilistic neural network classification results of MSE(1)∼(16).

Type	Recognized as					Sensitivity	Specificity
	A	B	C	D	E		
**A**	15	1	3	11	0	50%	90%
**B**	2	23	0	0	5	76.7%	93.3%
**C**	2	0	28	0	0	93.3%	95.8%
**D**	8	1	2	17	2	56.7%	89.2%
**E**	0	6	0	2	22	73.3%	94.2%
**Accuracy**	70%						

## Abbreviations

The following abbreviations are used in this manuscript:

AE	Approximate entropy
SE	Sample entropy
MSE	Multiscale sample entropy
HE	Hierarchical entropy
VMD	Variational mode decomposition
MPE	multiscale permutation entropy
EEMD	Ensemble empirical mode decomposition
SD	Standard deviation
SNR	Signal-to-noise ratio
